# Gut Microbiome Suffers from Hematopoietic Stem Cell Transplantation in Childhood and Its Characteristics Are Positively Associated with Intra-Hospital Physical Exercise

**DOI:** 10.3390/biology11050785

**Published:** 2022-05-21

**Authors:** Simona Ugrayová, Peter Švec, Ivan Hric, Sára Šardzíková, Libuša Kubáňová, Adela Penesová, Jaroslava Adamčáková, Petra Pačesová, Júlia Horáková, Alexandra Kolenová, Katarína Šoltys, Martin Kolisek, Viktor Bielik

**Affiliations:** 1 Department of Biological and Medical Science, Faculty of Physical Education and Sport, Comenius University in Bratislava, 814 69 Bratislava, Slovakia; simona.ugrayova@uniba.sk (S.U.); ivan.hric@uniba.sk (I.H.); libusa.kubanova@uniba.sk (L.K.); 2 Department of Pediatric Hematology and Oncology, Comenius University and National Institute of Children’s Diseases, Limbova 1, 833 40 Bratislava, Slovakia; peter.svec@gmail.com (P.Š.); jaroslava.adamcakova@gmail.com (J.A.); julia.horakova@nudch.eu (J.H.); alexandra.kolenova@nudch.eu (A.K.); 3 Department of Microbiology and Virology, Faculty of Natural Sciences, Comenius University in Bratislava, 842 15 Bratislava, Slovakia; sardzikova2@uniba.sk (S.Š.); katarina.soltys@uniba.sk (K.Š.); 4 Biomedical Center, Institute of Clinical and Translational Research, Slovak Academy of Sciences, 845 05 Bratislava, Slovakia; adela.penesova@savba.sk; 5 Department of Sports Educology and Sports Humanistic, Faculty of Physical Education and Sports, Comenius University in Bratislava, 814 69 Bratislava, Slovakia; petra.pacesova@uniba.sk; 6 Comenius University Science Park, Comenius University in Bratislava, 841 04 Bratislava, Slovakia; 7 Biomedical Center Martin, Jessenius Faculty of Medicine in Martin, Comenius University in Bratislava, 036 01 Martin, Slovakia; martin.kolisek@uniba.sk

**Keywords:** physical activity, leukemia, exercise, parenteral nutrition, CRP, *Enterococcus* spp.

## Abstract

**Simple Summary:**

Gut microbiome research has rapidly advanced with good perspectives for the diagnostics and/or therapy of a plethora of diseases, including metabolic and neurodegenerative diseases and various types of cancer. In this study, we examined the composition of the fecal microbiota of eligible children with acute lymphoblastic leukemia (ALL) after hematopoietic stem cell transplantation (HSCT). We found a negative effect of HSCT treatment on microbiome bacterial diversity and richness with microbial monodominance by pathogenic bacteria in pediatric ALL patients after 3 months. The changes in the gut microbiota were associated with systemic inflammation on day + 28. However, promisingly, we have observed an association between bacterial diversity and physical exercise during HSCT treatment. Our findings provide additional support for the importance of investigating the gut microbiome in children with cancer.

**Abstract:**

Gut microbiome impairment is a serious side effect of cancer treatment. The aim of this study was to identify the effects of hematopoietic stem cell transplantation (HSCT) treatment on gut microbiota composition in children with acute lymphoblastic leukemia (ALL). Fecal microbiotas were categorized using specific primers targeting the V1–V3 region of 16S rDNA in eligible pediatric ALL patients after HSCT (n = 16) and in healthy controls (Ctrl, n = 13). An intra-hospital exercise program was also organized for child patients during HSCT treatment. Significant differences in gut microbiota composition were observed between ALL HSCT and Ctrl with further negative effects. Plasma C-reactive protein correlated positively with the pathogenic bacteria *Enterococcus* spp. and negatively with beneficial bacteria *Butyriccocus* spp. or *Akkermansia* spp., respectively (rs = 0.511, *p* = 0.05; rs = −0.541, *p* = 0.04; rs = −0.738, *p* = 0.02). Bacterial alpha diversity correlated with the exercise training characteristics. Therefore, specific changes in the microbiota of children were associated with systemic inflammation or the ability to exercise physically during HSCT treatment.

## 1. Introduction

Hematopoietic stem cell transplantation (HSCT) in pediatric patients is used in the treatment of malignant and non-malignant disorders [1]. However, the toxicity of treatment as a consequence of chemotherapy, radiotherapy, and/or corticosteroid treatment induces known side effects and complications, including oral mucositis [2] systemic inflammation [3], weakness and fatigue in the skeletal muscle of survivors [4], lower mineral density [5], impaired cardiorespiratory fitness, muscle strength, and motor function [6,7]. Moreover, following HSCT, survivors can later develop metabolic syndrome and dyslipidemia [8]. Whereas considerable evidence exists concerning the effects of antibiotic treatment on the intestinal community in HSCT adult survivors [9], only limited research findings have been presented regarding the composition of the intestinal microbiota in children after HSCT [10,11,12,13]. However, gut microbiome impairment is another, no less serious, side effect found in HSCT pediatric patients [14]. Increasing evidence indicates that the intestinal microbiome is a promising tool for improving patient health [15]. The main purpose of this pilot study has been to determine microbiota diversity and the relative abundance of bacteria at each taxonomic level before and during HSCT treatment in pediatric patients and to identify clinical signs associated with specific changes in the gut microbiota.

Promising fitness and overall health benefits have been reported, even after a relatively short-term intra-hospital exercise training program in pediatric patients after bone marrow transplantation [16]. Unfortunately, most of the positive effects resulting from regular physical activity during cancer treatment are documented primarily in adult patients [17]. Therefore, another aim of this pilot study has been to find an association between intra-hospital physical exercises completed by children after HSCT and the structure of their microbiota. Our intention has been to create a structured exercise program carried out within a relatively small hospital transplant room (transplant box) with limited exercise equipment and strict hygienic precautions. Since enteral nutrition has been shown to be more effective in promoting intestinal microbial homeostasis compared with parenteral nutrition [18], our study has also focused on finding an association between gut microbiome and supportive nutritional treatment.

## 2. Materials and Methods

### 2.1. Subjects

Pediatric patients diagnosed with ALL were recruited from May 2018 to September 2021 at the Department of Pediatric Hematology and Oncology-Bone Marrow Transplant Unit (Faculty of Medicine at Comenius University and National Institute of Child Diseases). The pediatric patients (n = 16) and healthy controls (n = 13) were aged 7–19 years. The physical characteristics of the pediatric patients were height (159.23 ± 23 cm), weight (41.68 ± 21.14 kg), and BMI (17.14 ± 4.25 kg/m^2^). Healthy probands in the control group were selected to approximate the composition and data variance of physical characteristics of the oncological cohort. The demographic characteristics of the healthy subjects were height (144.09 ± 17.79 cm), weight (33.7 ± 15.65 kg), and BMI (15.67 ± 4.73 kg/m^2^). This study was carried out in conformity with the Principles of the Declaration of Helsinki for experiments involving human beings. Following the reading of the informed consent form, an explanation of the steps of the study, and discussions with the investigators, a signed informed consent allowing for the participation of their children in the study was obtained from all legal representatives (parents). The study was approved by the Ethics Committee of the National Institute of Child Diseases (NICD-25/4/18).

### 2.2. Intervention

**Figure 1:**
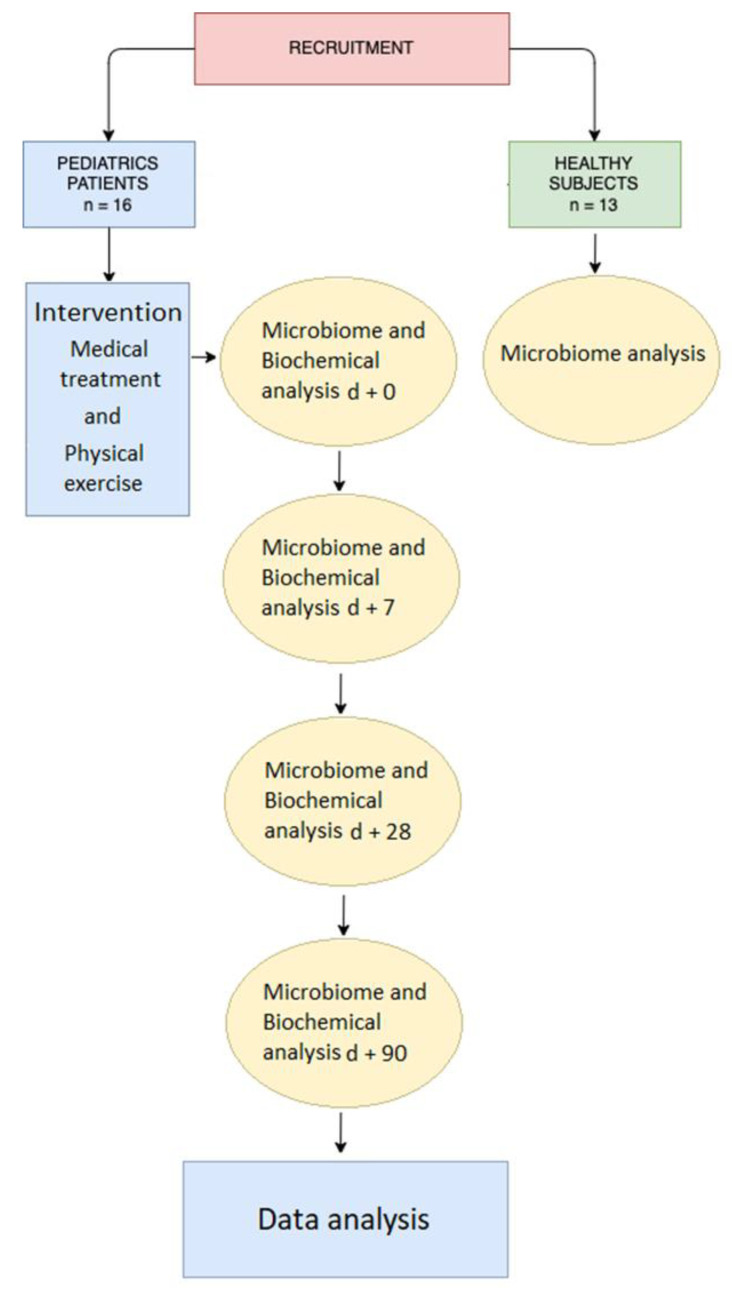
Diagram: Flowchart of the study.

A flowchart of the study is presented in Figure 1.

#### 2.2.1. Medical Treatment

The administration of antibiotic therapy was recorded for all patients. Routine prophylaxis was provided in accordance with unit policy: Intravenous Piperacillin/Tazobactam, Meropenem, Amikacin, Linezolid, and Teicoplanin at day + 0 until discharge or commencement of antibiotic treatment (Cefuroxime, Rifaximin, Sulfamethoxazole-Trimethoprim, or Cyprofloxacin) by mouth. Uniform standard anti-infective prophylaxis was provided to patients undergoing HSCT. Empirical, preemptive, or targeted anti-infectious therapy was performed with various antiviral (e.g., Aciclovir and Ganciclovir) and antifungal (e.g., Caspofungin, Mikafungin, and Flukonazol) agents, in accordance with commonly accepted strategies. The first step was total-body irradiation (TBI) combined with intensive chemotherapy treatment. TBI was carried out in six fractions over three consecutive days, namely at −3, −2, and −1, within a teletherapy unit using vertical beams. To intensify this TBI regime and to reduce relapse, we added a medium-dose treatment of Etoposide (VP16 40–60 mg/kg). Patients who were candidates for a reduced-intensity conditioning regime underwent treatment with Busulfan following Fludarabine at days 33, −2, and −1. Stem cell mobilization therapy included Filgrastim at 10 μg/kg per day (between days + 8 and + 28). Standard prophylaxis therapy for graft-versus-host disease (GvHD) involved Cyclosporine A (CsA) commencing at day −1, ATG—Thymoglobuline or ATG—Grafalon commencing at days −3, −2, and −1, Methotrexate (MTX) at 15 mg per meter squared (m^2^) of body size once on day + 1 and at 10 mg/m^2^ once per day on days + 3 and 6, and then Calcium Leucovorin at 5 mg starting 24 h after each dose of MTX on days + 2, 4, and 7. Some patients were given intravenous immunoglobulin (IVIg) therapy between days + 8 and + 28 to enable them to make enough antibodies to fight pathogens causing infections. The dosage of the drugs was calculated in accordance with the weight and health condition of the patients. The lengths of hospital stay (< or ≥30 days) were recorded from day + 0. Survival at day + 90 post-HSCT was noted.

#### 2.2.2. Physical Exercise

The individual training program offered during the hospital stay of the patients included 25–45 min of physical exercise, two times weekly, under the supervision of a certified sports trainer from the Faculty of Physical Education and Sports Comenius University. Familiarization between the trainers and pediatric patient took place 1 week before HSCT treatment. Exercise training occurred in the hospital transplant room (transplant box) under special hygienic precautions. The structure of the exercise program was developed to improve endurance and gradually re-build muscular strength. Training sessions were aimed at the large muscle groups with emphasis on correct technique. Special attention was paid to the squat as an exercise needed to provide basic needs (e.g., sitting, standing). The number of repetitions and series for each exercise was in the range of 10–15 repetitions and 2–3 series. In general, the exercises involved three modalities based on difficulty. The selection and modalities of the exercise respected the ability and condition of the patient. Each training session was recommended by the physician on duty in consultation with the senior physiotherapist from the Physiotherapy and Rehabilitation Department of NICD, as required.

### 2.3. Sample Collection

Fecal samples from the ALL HSCT patients were collected on the day of transplantation (d + 0) and consequently on d + 7, d + 28, and d + 90. Nurses were instructed with regard to the prevention of the contamination of samples during sample collection and were provided with DNA/RNA Shield Fecal Collection Tubes for fecal collection and the preservation of nucleic acids from stool specimens (ZymoResearch, Irvine, CA, USA). Samples were stored in the DNA/RNA Shield Fecal Collection Tubes at ambient temperature in the National Institute of Childhood Diseases Bratislava until they were delivered to the laboratory. Samples were then stored at −80 °C. Six patients had incomplete data (day + 7, n = 1; + 28, n = 2 and + 90, n = 3) due to missing or contamination of fecal samples.

### 2.4. Microbiome Analysis

The total DNA from the stool samples was extracted using the ZymoBiomics DNA/RNA mini kit (ZymoResearch Scientific, Irvine, CA, USA), in accordance with the manufacturer’s protocol. DNA was amplified using specific primers that targeted the V1–V3 region of 16S rDNA. Amplicons were used for the preparation of DNA libraries and sequenced using the Illumina MiSeq platform by 300-bp pair-end reads (Illumina, San Diego, CA, USA). Details of the procedure for DNA sample sequencing were as described by Hric et al. [19].

### 2.5. Illumina Data Processing

We analyzed the output of pair readings from Illumina MiSeq by the bioinformatics program Geneious. We adjusted the program sequences in accordance with the quality of bases Q >25 by trimming 3’ends-post-trim: 50 bp. The readings were merged through the merge pair reads using FLASH with a minimum overlap of 50 bp and a single overlap of 150 bp. Subsequently, we analyzed the data by means of the online tool SILVAngs [20].

### 2.6. Biochemical Analysis

Biochemical parameters were measured on a daily basis in the Department of Laboratory Medicine (National Institute of Childhood Diseases Bratislava, Slovakia). C-reactive protein (CRP) levels were determined using in vitro immune kinetic enzymatic assay in human serum on layered reagent media with reflectometry measurements on a Vitros 4600 analyzer (Ortho-Clinical Diagnostics, Inc., Rochester, NY, USA). Procalcitonin (PCT) levels were measured by an electrochemiluminescence immunoassay (ECLIA) in human serum for quantitative determination in a Cobas e411 immunoassay analyzer (Roche Diagnostics GmbH, Mannheim, Germany).

### 2.7. Statistical Analysis

Statistical analyses were carried out using the SPSS 21.0 program for Windows (SPSS, Inc., Chicago, IL, USA). Data normality was checked by the Shapiro-Wilk test. An independent *t*-test was conducted to achieve a comparison of nonparametric data of gut microbiome between the control (Ctrl) group and each ALL HSCT group. Furthermore, data from pediatric ALL HSCT survivor groups at d + 0, d + 7, d + 28, and d + 90 were subjected to Welch and Brown-Forsythe versions of the one-way ANOVA. Spearman’s rank correlation coefficient was used to assess the relationship between variables (gut microbiome and training variables). The significance level of *p* < 0.05 was applied. ClustVis was used to visualize multidimensional data by means of principal component analysis (PCA) [21].

## 3. Results

### 3.1. Fecal Microbiota

From the collected 68 fecal specimens, we detected 14 bacterial phyla in the Ctrl group. In the samples from ALL HSCT patients, we found 17 families on d + 0, 15 on d + 7, 10 on d + 28, and 12 on d + 90. Phyla with the highest relative abundance in the Ctrl group were the Firmicutes (64.87%), Bacteroidota (29.57%), Actinobacteriota (2.56%), and Proteobacteria (2.40%). The phyla Verrucomicrobiota, Cyanobacteria, Fusobacteriota, Desulfobacterota, Campylobacterota, Patescibacteria, Fibrobacterota, Synergistota, Spirochaetota, and Gemmatimonadota were detected with an abundance of less than 1%. In ALL patients, the predominant phyla were the Firmicutes (d + 0—74.12%; d + 7—72.67%; d + 28—83.17%; d + 90—83.91%), Bacteroidota (d + 0—13.18%; d + 7—5.09%; d + 28—6.89%; d + 90—4.59%), Proteobacteria (d + 0—9.63%; d + 7—17.19%; d + 28—8.73%; d + 90—11.16%), Actinobacteriota (d + 0—1.44%; d + 7—3.17%; d + 28—1.18%; d + 90—0.31%), and Verrucomicrobiota (d + 0—1.26%; d + 7—1.84%; d + 28—0.01%; d + 90—0.02%). Cyanobacteria, Fusobacteriota, Desulfobacterota, Campylobacterota, Patescibacteria, Synergistota, Spirochaetota, Acidobacteriota, Myxococcota, Bdellovibrionota, WPS-2, and Deinococcota were present with an abundance of less than 1%.

In the Ctrl group, we determined 84 taxons at the family level. In the samples from ALL HSCT patients, we found 110 families on d+0, 98 on d + 7, 85 on d + 28, and 87 on d + 90. The dominant families in the Ctrl group were the Lachnospiraceae (30.98%), Ruminococcaceae (21.77%), Bacteroidaceae (12.49%), Prevotellaceae (10.21%), Oscillospiraceae (5.15%), and Rikenellaceae (3.70%) followed by families Coriobacteriaceae, Sutterellaceae, Christensenellaceae, Tannerellaceae, Peptostreptococcaceae, and Clostridiaceae. The series of dominant families were different in the ALL patients, e.g., Enterococcaceae (d + 0—19.36%; d + 7—33.83%; d + 28—45.02%; d + 90—52.08%), Lachnospiraceae (d + 0—24.89%; d + 7—15.54%; d + 28—0.48%; d + 90—9.69%), Bacteroidaceae (d + 0—12.38%; d + 7—0.31%; d + 28—6.85%; d + 90—4.30%), Enterobacteriaceae (d + 0—9.01%; d + 7—10.39%; d + 28—7.65%; d + 90—7.77%), Ruminococcaceae (d + 0—6.28%; d + 7—2.59%; d + 28—0.19%; d + 90—3.05%), Staphylococcaceae ( d + 0—0.53%; d + 7—5.81%; d + 28—14.41%; d + 90—11.06%), Streptococcaceae (d + 0—1.89%; d + 7—4.84%; d + 28—9.26%; d + 90—2.32%), and Erysipelotrichaceae (d + 0—5.39%; d + 7—3.51%; d + 28—0.15%; d + 90—0.12%) followed by families Xanthomonadaceae, Akkermansiaceae, Oscillospiraceae, and Carnobacteriaceae. On the other hand, the family Lactobacillaceae was dominant only on d + 28 (11.29%) and, in other groups of samples, was detected with an abundance of less than 1%.

**Figure 2:**
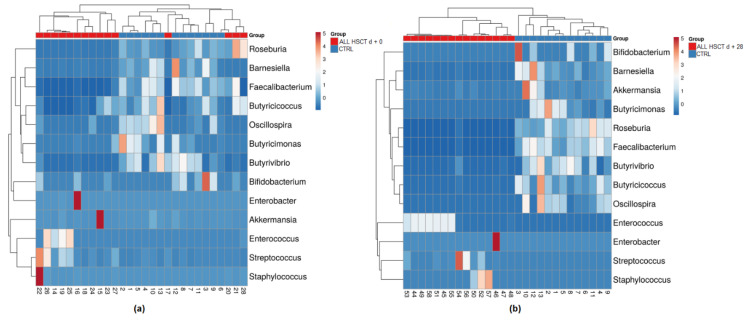
Microbial composition of significantly (*p* < 0.05) distinct bacterial genera within the healthy control group (Ctrl) and pediatric ALL patients after HSCT treatment at day + 0 (**a**), and at day + 28 (**b**). Selected rows are centered; unit variance scaling is applied to rows. Imputation is used for missing value estimation. Both rows and columns are clustered by means of correlation distance and average linkage. Numbers represent samples visualized by heat mapping.

In total, 532 genera were identified, of which 228 genera were in the Ctrl group. In the pediatric patients, 296 genera were detected on d + 0, 244 on d + 7, 193 on d + 28, and 217 on d + 90. The 10 most abundant genera identified in Ctrl group were the *Faecalibacterium, Bacteroides, Prevotella_9, Blautia, Alistipes, Ruminococcus, Subdoligranulum, Roseburia, Lachnospiraceae UCG-004,* and *Agathobacter*. The five most abundant genera in the fecal samples of the pediatric patients were collection-day-dependent: d + 0 (*Enterococcus, Bacteroides, Enterobacter*, (*Ruminococcus) gnavus group, Lachnoclostridium*), d + 7 (*Enterococcus, Lachnoclostridium, Stenotrophomonas, Staphylococcus, Enterobacter*), d + 28 (*Enterococcus, Staphylococcus, Streptococcus, Bacteroides, Enterobacter*), and d + 90 (*Enterococcus, Staphylococcus, Bacteroides, Klebsiella, Lachnoclostridium*) (Figure 2). Complete populations of bacteria are reported in Supplementary File S1.

### 3.2. Bacterial Diversity

**Figure 3:**
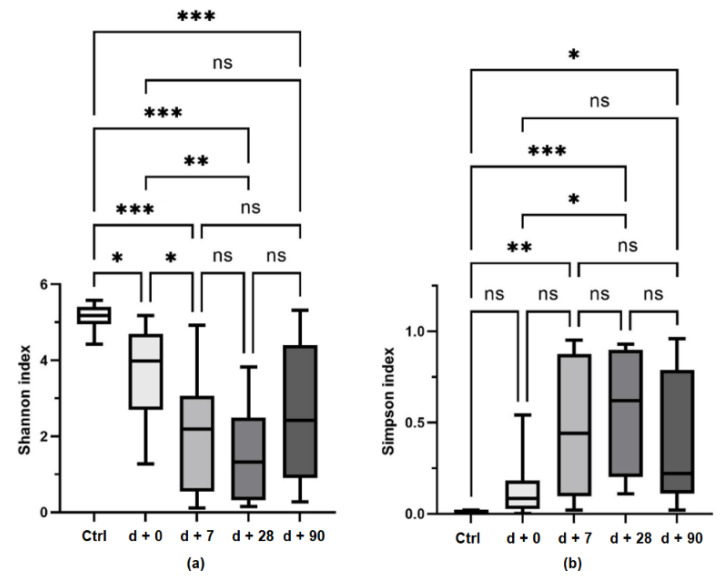
Alpha diversity of gut microbiome in healthy subjects (n = 13) and in pediatric ALL survivors at days + 0, + 7, + 28, and + 90 after HSCT treatment. (**a**) Shannon index; (**b**) Simpson index. * *p* < 0.05, ** *p* < 0.01, and *** *p* < 0.001, ns: non-significant.

**Figure 4:**
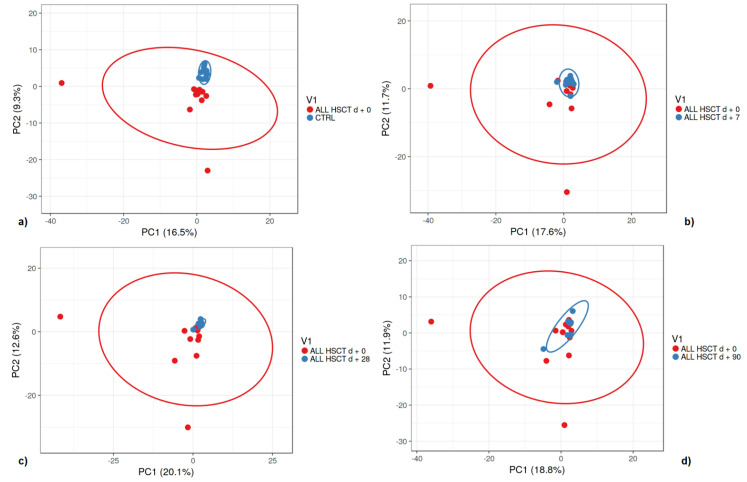
Beta diversity of analyzed samples represented by all genera OTUs in pediatric ALL patients after HSCT treatment at day + 0 and healthy controls (Ctrl) (**a**), day + 7 (**b**), day + 28 (**c**), and day + 90 (**d**) visualized by PCA. SVD with imputation was used to calculate principal components. X and Y axes show principal component 1 and principal component 2 explaining 16.5% and 9.3% of the total variance, respectively, in (**a**) 17.6% and 11.7%, respectively; in (**b**) 20.1% and 12.6%, respectively; in (**c**) 18.8% and 11.9%, respectively; in (**d**). Prediction ellipses are drawn in order that, with a probability of 0.95, a new observation from the same group will fall inside the ellipse (N = 28 data points).

A comparison of the gut microbiome alpha diversity between healthy controls and pediatric patients and the shift of bacterial diversity during HSCT treatment is presented in Figure 3. A comparison of gut microbiome beta diversity between healthy controls and pediatric patients is presented in Figure 4.

### 3.3. Clinical Signs and the Microbiome

Following HSCT treatment, parenteral nutrition was administered to patients through the venous system. However, some patients were able to eat some solid food from d + 0. One patient received only parenteral nutrition until d + 91. The total number of days on parenteral nutrition support during 3 months after HSCT individually ranged from 25–91. Patient demographics and clinical characteristics are detailed in Table 1, and parenteral nutrition details are presented in Supplementary File S2. Near significant positive association was found between the total number of days on parenteral nutrition and the abundance of unclassified *Enterobacter* species (spp.) d + 28 (rs = 0.579, *p* = 0.06). The transition from parenteral support to per. os. nutrition ranged from d + 32 to d + 119. A significant positive association was observed between the day of transition from parenteral support to per. os. nutrition and procalcitonin (PCT) levels at d + 7 and d + 28, respectively (rs = 0.532, *p* = 0.05; rs = 0.652, *p* = 0.02).

The shortest hospital stay lasted until d + 32 and the longest until d +122. The length of hospital stay was significantly correlated to pathogenic bacteria *Enterobacter* spp. (rs = 0.552, *p* = 0.03). Furthermore, a significant negative association was observed between the plasma C-reactive protein (CRP) d + 28 and OTUs d + 28, *Butyriccocus* spp. d + 28 or *Akkermansia* spp. d + 90 (rs = −0.668, *p* = 0.01; rs = −0.541, *p* = 0.04; rs = −0.738, *p* = 0.02). The abundance of *Enterococcus* spp. d + 28 was positively correlated with CRP d + 28 (rs = 0.511, *p* = 0.05).

### 3.4. Exercise Training

With regard to the start of the exercises during the early stages after HSCT, we had to respect each patient’s condition (e.g., exercise sessions could be contra-indicated if the patient exhibited fever, persistent weakness, etc.). In general, the patients were able to commence physical training at the earliest by 7–10 days after HSCT treatment. The exercise program was influenced by the first and second wave of the COVID-19 pandemic, since the Government of the Slovak Republic declared a state of emergency, with the result that physical trainers were not allowed to enter hospitals. Nevertheless, the patients were able to carry out their exercise program under supervision via the online communication platform Microsoft Teams. Some of the planned exercise sessions had to be omitted due to the poor clinical condition of some of the patients, and therefore, the characteristics of the training program (number of sessions and total volume of exercise) varied between patients. Total intra-hospital exercise units completed by patients ranged from 1 to 17. A significant positive association was observed between the total number of exercise sessions during the hospital stay and the alpha diversity measured by the Shannon index on d + 90 (rs = −0.794, *p* = 0.006). In accordance with this finding, the Simpson index was negatively correlated with the number of sessions (rs = −0.772, *p* = 0.009).

The total net amount of intra-hospital exercise ranged from to 10 to 600 min. A significant positive association was found between the total amount of exercise (min) during the hospital stay and the Shannon index on d + 90 (rs = −0.763, *p* = 0.01). On the other hand, the Simpson index was negatively correlated with the number of sessions (rs = −0.815, *p* = 0.004). Furthermore, a significant positive association was detected between the total amount of exercise and the number of OTUs on d + 90 (rs = 0.726, *p* = 0.017).

## 4. Discussion

In the present study, we report the composition of the gut microbiome in children with acute lymphoblastic leukemia (ALL) treated by hematopoietic stem cell transplantation (HSCT). We hypothesized that differences would be apparent in bacterial richness between healthy controls and child patients, with further negative effects on the gut microbiome during the 3 months after HSCT. Moreover, we hypothesized that specific changes in the microbiota would coincide with systemic inflammation markers or the ability to undergo physical exercise during HSCT treatment.

We used a structured exercise program with emphasis on functional mobility and the gradual building of strength and endurance.

Our main findings demonstrated a negative effect of cancer treatment on bacterial diversity and richness as measured by the alpha diversity between patients and healthy controls. Furthermore, we found an increase in the relative abundance of pathogenic bacteria (*Enterobacter* spp., *Klebsiella* spp.) during the 3 months after HSCT. Some pathogenic bacteria were positively associated with sensitive markers of bacterial infections. In addition, we found a promising association between physical exercise variables (total volume and number of session) and bacterial diversity (Shannon and Simpson index).

Loss of intestinal microbiome diversity is well documented in pediatric ALL patients compared with healthy controls [12,22]. Although the mechanism of impairment of the gut microbiome by chemotherapeutics remains unknown, the early microbiome shifts at the onset of therapy are thought to be related to chemotherapeutic and corticosteroid treatment [23,24,25]. One of the most common negative effects of chemotherapy and radiotherapy is intestinal mucositis [26]. Intestinal mucositis developing during chemotherapy coincides with plasma CRP levels and may be reduced by butyrate-producing bacteria [25]. Interestingly, we found a significant negative association between plasma CRP d + 28 and *Butyriccocus* spp. d + 28 or *Akkermansia* spp. d + 90. However, in patients who have survived HSCT, early microbiome shifts and a loss of gut microbiome diversity have also been related to antibiotic treatment [27,28].

Here, we report the differences in alpha diversity between controls and child HSCT survivors at days + 0, + 7, + 28, and + 90. Almost all of our patients had been previously diagnosed with ALL and unsuccessfully treated by chemotherapy, corticosteroids, and antibiotics. Despite the possible prior disruption of the microbiome in these patients, we observed indications of a loss of intestinal microbiome richness and diversity between days + 0 and + 28 after HSCT treatment. This outcome might be explained by the higher dose of antibiotic treatment in the early phases after HSCT treatment. We further report a significant difference in alpha diversity between healthy controls and patients at day +90, but no significant differences between days + 0 and + 90 in the patient group. Our data show some positive trends in the gut microbiome shift between days + 30 and + 90. Interestingly, as reported previously, stool samples collected from survivors (aged under 19 years) at least 1 year after the completion of HSCT did not differ in alpha or beta diversity from their sibling controls [29]. Similarly, data from 32 pediatric and adolescent ALL patients have revealed that gut microbial diversity tends to stabilize after 1-year post-chemotherapy [30]. However, the authors of the latter study admit the persistence of gut microbial dysbiosis induced by the abundance of mucolytic Gram-positive anaerobic bacteria, specifically by *Ruminococcus gnavus* and *Ruminococcus torques* [30]. As shown in another investigation, the shift in gut microbiome composition with microbial monodominance (e.g., Enterobacteriaceae or *Enterococcus* spp.) precedes and might lead to a higher risk of bacteremia in HSCT patients [31]. Data from a retrospective cohort study of 7128 adult and pediatric patients who had undergone HSCT exhibited an association of *Enterococcus* bacteremia with lower overall survival and increased non-relapse mortality [32]. Here, we report dysbiosis induced by unclassified *Enterococcus* species (spp.) and the family *Enterococcaceae* as the dominant bacteria in our group of pediatric patients between +7 and +90 days following HSCT. In addition, the lengths of the hospital stay of our patients were significantly correlated to pathogenic bacteria *Enterobacter* spp. Furthermore, we report a positive association between *Enterococcus* spp. d + 28 and CRP d + 28. These findings are in accordance with a recent study on children newly-diagnosed with ALL [25]. In general, an increased abundance of *Enterococcaceae* follows chemotherapy treatment in childhood ALL [23].

Recently, Andersen et al. [33] have reported a difference in the microbiome between patients receiving enteral and parenteral nutrition following allogeneic hematopoietic progenitor cell transplantation. Patients with minimal oral food intake with parenteral support have lower microbial diversity [33]. Although only nearly significant (*p* = 0.06), we have found a positive association between the total number of days on parenteral nutrition and abundance of *Enterobacter* spp. at d + 28. In particular, enteral nutrition more positively affects the gut microbiome composition compared with parenteral nutrition [18]. Enteral nutrition in pediatric patients undergoing HSCT is a prerequisite for a better recovery of gut microbiome diversity [34]. Furthermore, the preference of enteral nutrition seems to result in a reduced incidence of blood stream infections [35] and of acute GvHD [36] and is associated with several benefits in HSCT pediatric patients [37]. With regard to the transition from parenteral to solid food, we found a positive association with procalcitonin (PCT) at d + 7 and d + 28. PCT, the peptide precursor of calcitonin, is a sensitive marker of severe bacterial infections in neutropenic children [38]. However, the individual regime and timing of supportive nutrition provided to patients after cell transplantation is characterized by great variation [39].

However, promisingly, we have observed a favorable significant positive association on day + 90 between training variables (frequency and volume of exercise) and the Shannon index. We further report an inverse correlation between training variables and the Simpson index. Moreover, the number of OTUs on day + 90 was significantly correlated with the total amount of intra-hospital exercise. Despite the proven positive results achieved following physical exercise in adult cancer patients and the rational reasons presented, limited scientific evidence of benefit exists for these effects in childhood leukemia [40,41,42]. Although the treatment of childhood leukemia is known to impair endurance, strength, and functional mobility [43], outcomes following exercise in children patients are not as convincing as those in adults. Generally, exercise programs for cancer patients presented in the literature focus on endurance and propose the gradual re-building of strength [44]. The authors of a multicenter randomized controlled trial have found no effects of a short-term exercise program on the physical fitness and psychosocial function of pediatric cancer patients compared with children who received the usual care [45]. However, exercise training is considered as a safe and, at least partly, effective option for the improvement of endothelial function and reduction of the waist circumference and the waist-to-hip ratio as well as for an increase in physical activity levels in childhood cancer survivors [46]. Weak results have mostly been associated with poor adherence to exercise interventions with a 25% to 100% variation and/or insufficient exercise dose [47].

The cardiorespiratory fitness of pediatric HSCT survivors can be impaired years after the completion of their treatment [7]. Supportive research of physical exercise in children during HSCT has shown positive effects on weight, body mass index, body fat, and fat-free mass [48], on the quality of life and fatigue symptoms [42], on children’s pain [49], and on decreasing the risk of infection associated with HSCT [50]. Unfortunately, little information is available describing exercise training benefits on the intestinal microbiome in children with cancer, and especially in those after HSCT.

Our findings provide additional support for the importance of investigating the gut microbiome in children with cancer. We assume that the children undergoing less training are those with a poorer clinical condition, needing more parenteral nutrition support, and with a lower bacterial diversity and richness. Despite the positive correlation between training variables and bacterial alpha diversity, we suggest that a better clinical condition allows the children to exercise more. Our study further provides pilot data for future exercise intervention research in pediatric ALL HSCT survivors and contributes to important considerations with regard to the feasibility of intra-hospital physical exercise. Due to the limited number of children and adolescents with ALL treated yearly by HSCT in Slovakia, a significantly larger (European) study population with a similar specific diagnosis is needed to establish the benefits of exercise training on treatment prognosis in these patients. In the future, a greater uptake of intra-hospital exercise training and physical activities might improve the applicability and the effects of the program on clinical outcomes.

## 5. Conclusions

This study has established the negative effect of cancer treatment on bacterial diversity and richness in pediatric HSCT patients. The relative abundance of beneficial bacteria decreases from day + 0 to day + 90 after HSCT. Furthermore, we report a shift in the gut microbiome composition, with a microbial monodominance by unclassified *Enterococcus* species (spp.) and family Enterococcaceae during the 3 months after HSCT. These specific changes in the microbiota are associated with systemic inflammation on day + 28. However, promisingly, in this pilot study, we have observed an association between alpha bacterial diversity and training variables during HSCT treatment. We assume that the pediatric patients exhibiting a better clinical condition and more favorable gut microbiome can exercise more. Further research is required to confirm whether intervention involving physical exercise influences clinical outcomes through effects on the gut microbiota.

## Figures and Tables

**Table 1:** Patient demographics and clinical characteristics.

	Conditioning		
	TBI (n = 6)	FB (n = 10)	*p*
Demographics and clinical characteristics at baseline			
Female (n)	3	3	
Primary diagnosis			
Acute leukemia (n)	6	10	
Donor type			
Volunteer unrelated (n)	4	8	
Sibling (n)	2	2	
Clinical outcomes			
Parenteral nutrition (days)	32 (25–48)	60 (45–90)	0.05
Per. os. (days)	59 (30–66)	31 (0–47)	0.03
Length of hospital stay from day + 0	47 (29–96)	92 (62–122)	0.01
<30 days	0	0	
≥30 days	6	10	
Developed GvHD			
aGvHD (n)	2	7	
cGvHD (n)	0	0	
Occurrence of GvHD (day)	32 (23–42)	31 (13–55)	ns
Duration of GvHD (days)	22 (18–26)	42 (29–78)	ns
Survived to day + 90	5	10	

Data are reported as the average, minimum, and maximum for categorical data. TBI: Total body irradiation; FB: Fludarabine + Busulfan; aGvHD: Acute graft-versus-host disease (occurring within 100 days); cGvHD: Acute graft-versus-host disease (after 100 days); Per. os.: Peroral nutrition; n: Number of patients; *p*: *p*-value; ns: non-significant.

## Data Availability

Results of all analyses are included in this published article. The datasets generated and/or analysed during the current study are available from the corresponding author on reasonable request.

## Supplementary Materials

The following are available online at https://www.mdpi.com/article/10.3390/biology11050785/s1. File S1: Average relative abundance of gut bacteria at different taxonomic levels. File S2: Individual daily calories administered by parenteral nutrition and example of nutritional information of parenteral diet at oncology patient per day.
